# Retraction: A highly concise and practical route to clavaminols, sphinganine and (+)-spisulosine *via* indium mediated allylation of α-hydrazino aldehyde and a theoretical insight into the stereochemical aspects of the reaction

**DOI:** 10.1039/c9ra90005e

**Published:** 2019-02-01

**Authors:** Menaka Pandey, Partha Sarathi Chowdhury, Achintya Kumar Dutta, Pradeep Kumar, Sourav Pal

**Affiliations:** Division of Organic Chemistry, CSIR-NCL (National Chemical Laboratory) Pune 411008 India pk.tripathi@ncl.res.in +91 02025902050; Physical Chemistry Division, CSIR-NCL (National Chemical Laboratory) Pune 411008 India

## Abstract

Retraction of ‘A highly concise and practical route to clavaminols, sphinganine and (+)-spisulosine *via* indium mediated allylation of α-hydrazino aldehyde and a theoretical insight into the stereochemical aspects of the reaction’ by Menaka Pandey *et al.*, *RSC Adv*., 2013, **3**, 15442–15448.

We, the named authors, hereby wholly retract this *RSC Advances* article due to inappropriate alterations made to the NMR spectra presented in the Electronic Supporting Information (ESI) accompanying this article. A number of white boxes were added on top of the NMR spectra, which can be selected and removed to reveal obscured peaks.

The affected spectra are:

^13^C NMR spectra for 12, 13, 21, 1a, 1b, 1c, 2, and 3.

^1^H NMR spectra for 10, 11, 12, 13, 17, 18, 1a, 1b and 1c.

The Director of the CSIR-National Chemical Laboratory has confirmed that archive copies of some of the original spectra in question have been located and preliminary indication is that there was manipulation of the spectra.

However, corresponding author Pradeep Kumar has confirmed that Sourav Pal and Achintya Kumar Dutta did not participate in the collection of NMR data for this article.

This retraction supersedes the information provided in the Expression of Concern related to this article.

Signed: Menaka Pandey, Partha Sarathi Chowdhury, Achintya Kumar Dutta, Pradeep Kumar and Sourav Pal

Date: 18^th^ January 2019

Retraction endorsed by Andrew Shore, Executive Editor, *RSC Advances*

## Supplementary Material

